# Supplementary material for: Transcriptional Differences in Lipid-Metabolizing Enzymes in Murine Sebocytes Derived from Sebaceous Glands of the Skin and Preputial Glands
Source: Int J Mol Sci. 2021 Oct 27;22(21):11631. doi: 10.3390/ijms222111631 (PMC8584257; doi:10.3390/ijms222111631)
Supplement: Supplementary file 1 [file ijms-22-11631-s001.zip › ijms-1429715-supplementary.pdf]

International Journal of
Molecular Sciences

MDPI

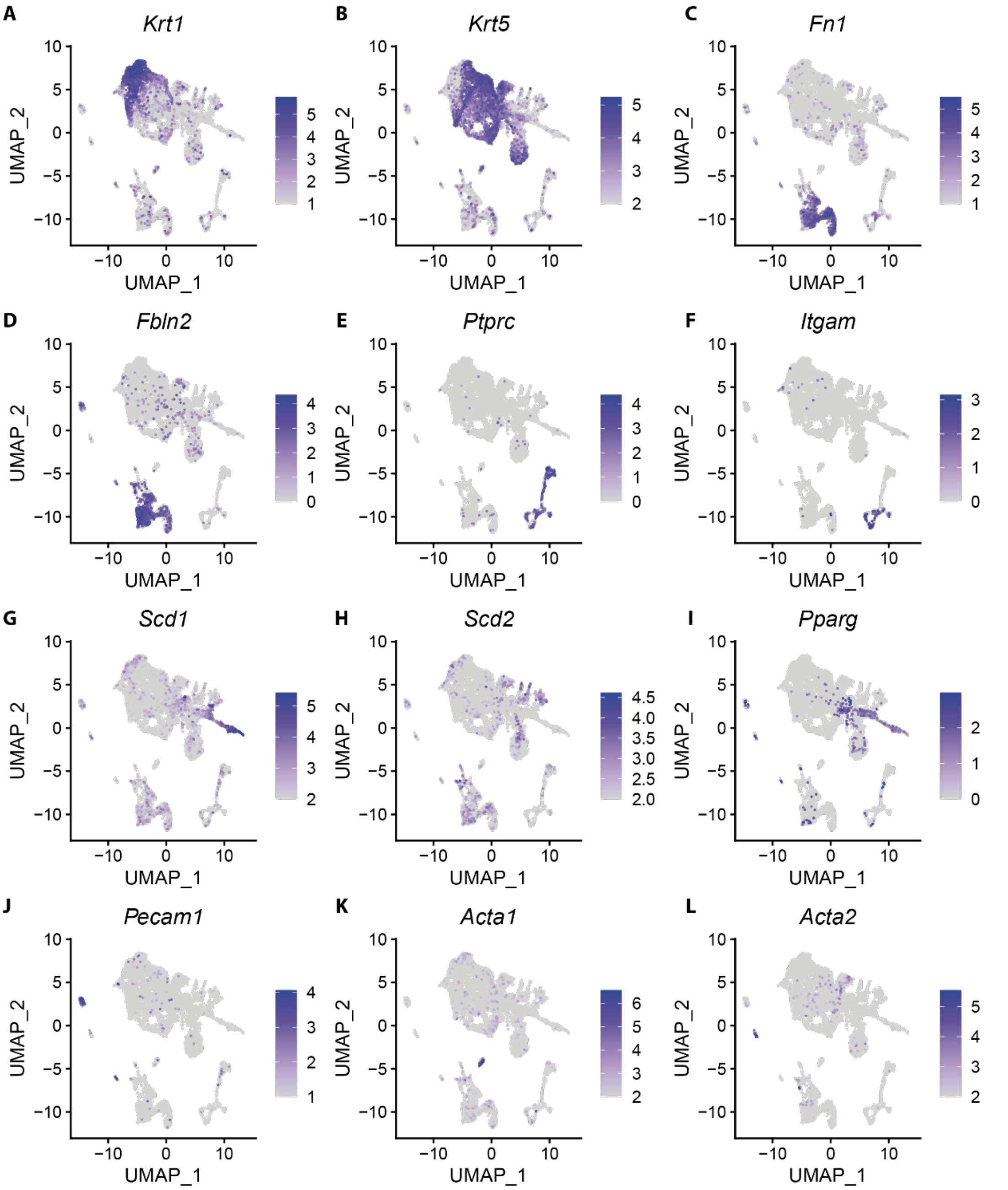

Figure S1. Cell Population definition based on clustermarker. Cell populations were identified as keratinocytes by (**A**) keratin 1 (*Krt1*) and (**B**) keratin 5 (*Krt5*), fibroblasts by (**C**) fibronectin (*Fn1*) and (**D**) fibulin-2 (*Fbln2*), immune cells by (**E**) CD45 (*Ptprc*) and (**F**) CD11b (*Itgam*), sebocytes by (**G**) stearoyl-CoA desaturase-1 (*Scd1*), (**H**) stearoyl-CoA desaturase-2 and (**I**) peroxisome proliferator activated receptor gamma (*Pparg*), endothelilal cells by (**J**) platelet and endothelial cell adhesion molecule 1 (*Pecam1*), muscle cells by (**K**) actin alpha 1 (*Acta1*) and smooth muscle cells by (**L**) actin alpha 2 (*Acta2*). In Feature Plots the normal log expression of the respective gene is mapped on the UMAP-Plot

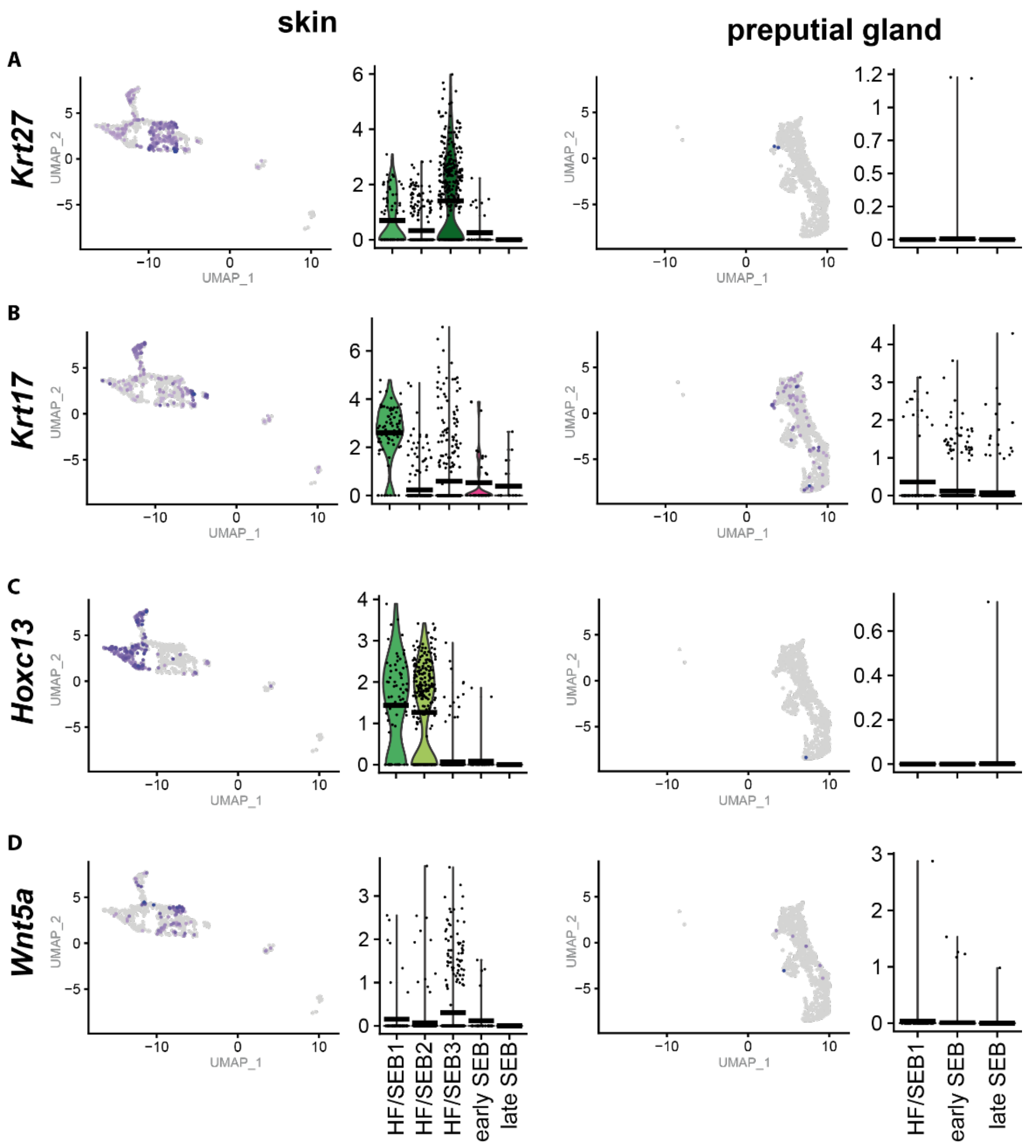

Figure S2. HF-associated gene expression in SEB. Featureplot and violin plot showing gene expression levels of HF-associated genes (**A**) keratin 27 (*Krt27*), (**B**) keratin 17 (*Krt17*), (**C**) homebox c13 (*Hoxc13*) and (**D**) Wnt family member 5A (*Wnt5a*). Left panel showing sSEB, right panel showing pSEB. Violin-plots show gene expression levels and crossbar of violin-plots depicts mean expresion value. Vertical lines show maximum expression. Width of violins represents the frequency of cells at the corresponding expression levels. In Feature Plots the normal log expression of the respective gene is mapped on the UMAP-Plot

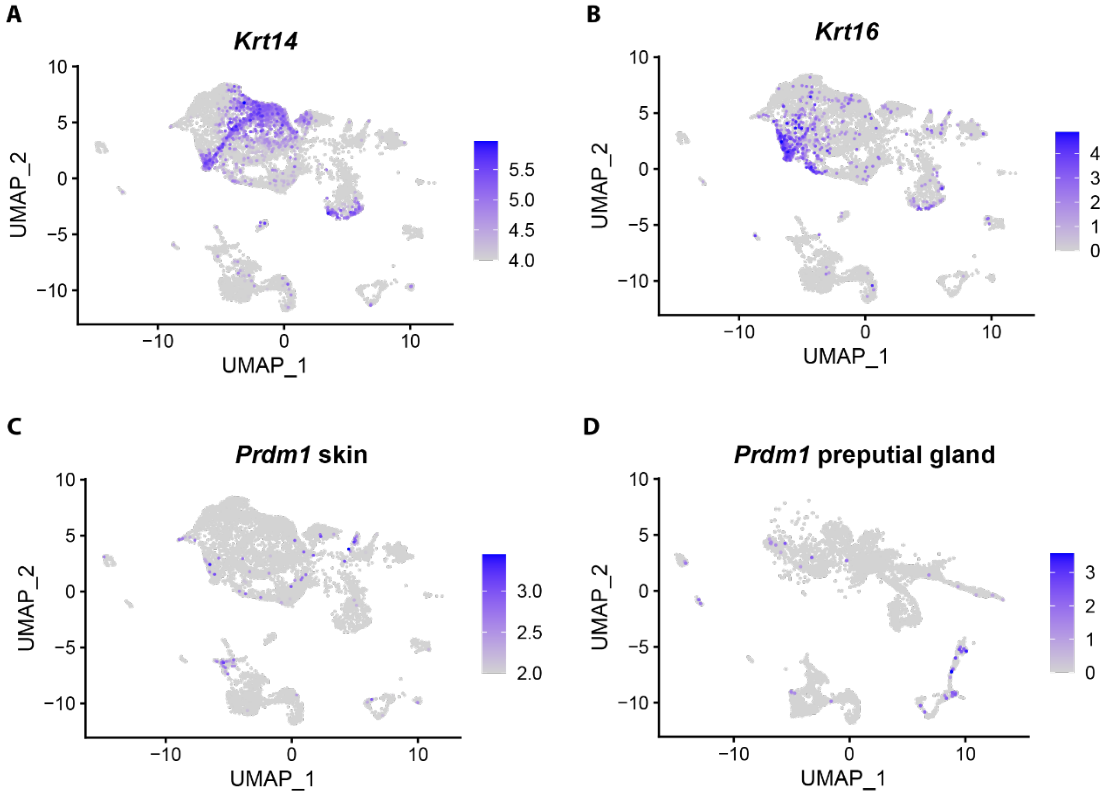

Figure S3. HF/Diff KC cluster marker and Blimp1 expression. Featureplots showing (**A**) keratin 14 (*Krt14*), (**B**) keratin 16 (*Krt16*) expression in skin. *Blimp1* (*Prdm1*) expression is shown for (**C**) skin and (**D**) preputial gland samples. In Feature Plots the normal log expression of the respective gene is mapped on the UMAP-Plot
